# High-Load Borage Oil Nanoemulsion Development via Polyol-Free D‑Phase Emulsification

**DOI:** 10.1021/acsomega.5c11660

**Published:** 2026-01-30

**Authors:** Jéssica Fagionato Masiero, Jonnatan Julival Santos, Andriéli Bacega, Enzo Boniconte Santomartino, Luiza de Oliveira Macedo, Geraldo José Arantes, Raimar Löbenberg, Gabriel Lima Barros de Araújo, Kelly Ishida, Nádia Araci Bou-Chacra

**Affiliations:** † Department of Pharmacy, Faculty of Pharmaceutical Sciences, University of São Paulo, Avenida Professor Lineu Prestes 580, Cidade Universitária, CEP: 05508-000 São Paulo, São Paulo, Brazil; ‡ Department of Fundamental Chemistry, Institute of Chemistry, University of Sao Paulo, Avenida Professor Lineu Prestes 748, Cidade Universitária, CEP: 05508-000 São Paulo, São Paulo, Brazil; § Department of Microbiology, Institute of Biomedical Sciences, 28133 University of São Paulo, Avenida Professor Lineu Prestes 1374, Cidade Universitária, CEP: 05508-000 São Paulo, São Paulo, Brazil; ∥ R&D Director, Alivira Animal Health and Nutrition, Avenida Espanha, 1025 Tibery, CEP: 38405-048 Uberlândia, Minas Gerais, Brazil; ⊥ Faculty of Pharmacy and Pharmaceutical Sciences, University of Alberta, T6G 2E1 Edmonton, Alberta, Canada

## Abstract

This study presents the development of borage oil nanoemulsions using the D-phase emulsification (DPE) method, notably excluding alkyl polyols. Utilizing polysorbate 80 as the surfactant, the method achieves high oil concentrations (up to 50% w/w) with minimal surfactant use, obviating the need for hydrophilic–lipophilic balance adjustments. A Box–Behnken design analyzed the effects of borage oil, surfactant, and initial water concentrations on particle size and stability. Initial water concentration significantly reduced the average hydrodynamic diameter (AHD), with optimized formulations showing spherical droplets of 300–400 nm, narrow size distributions (PdI < 0.3), and robust zeta potential (←20 mV), maintaining physical stability for 12 months. Microscopy, X-ray diffraction, and thermal analyses affirmed the amorphous or liquid crystalline state of the formulations. Scale-up to 1 kg retained similar physicochemical properties. The in vivo toxicity assessment in the *Galleria mellonella* model indicated no significant toxicity, highlighting the formulation’s safety. This scalable, solvent-free approach offers insights into the influence of key formulation variables, potentially advancing its application in pharmaceuticals.

## Graphical abstract

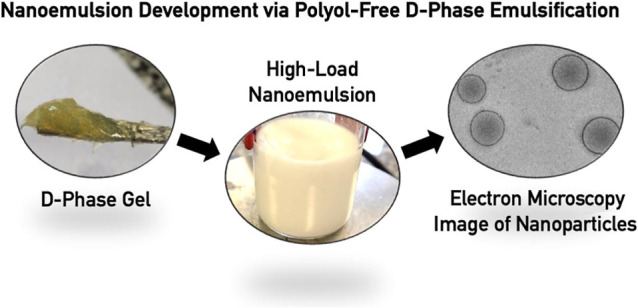

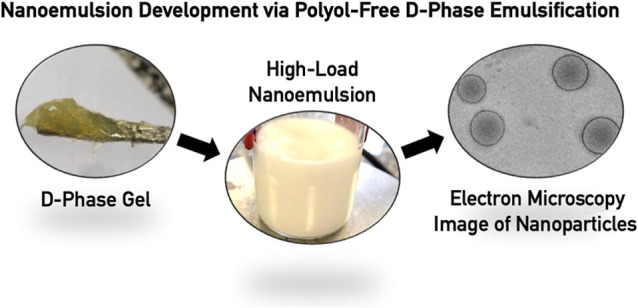

## 1 Introduction

Nanoemulsions, in particular, represent a specialized type of emulsion with particle sizes ranging from 50 to 1000 nm.^1–3^ Their modified-release properties and efficacy as drug delivery systems make them valuable for pharmaceutical formulations.^4,5^ However, their small particle size renders highly prone to instability phenomena, such as aggregation. Consequently, the choice of an appropriate preparation technique is critical, as the stability and particle size of nanoemulsions are significantly influenced by the preparation method.^6^

Nanoemulsions can be prepared using high or low-energy approaches. High-energy techniques, such as high-pressure homogenization and ultrasonication, employ intense mechanical forces to create large interfacial areas and generate nanometric droplets.^7^ While effective, these methods may compromise the integrity of bioactive molecules and require significant energy input.^8^ In contrast, a low-energy nanoemulsion is a finely dispersed system comprising two immiscible liquids, typically oil and water, where one is distributed within the other as nanometer-sized droplets. This type of nanoemulsion is achieved through methods such as phase inversion temperature (PIT), D-phase emulsification, or spontaneous emulsification, which do not require significant mechanical energy input. These processes leverage on the precise formulation and careful selection of surfactants, often incorporating vegetable oils, to effectively reduce interfacial tension and promote the self-assembly of nanosized droplets. This results in a stable and efficient system for drug delivery, enhancing solubility and bioavailability of active ingredients.^9^

The D-phase emulsification (DPE) method, introduced by Sagitani in the 1980s, offers advantages over conventional phase inversion techniques. It facilitates the formation of fine emulsions with high oil concentrations while requiring relatively low amounts of surfactants. Unlike phase inversion composition (PIC) and phase inversion temperature (PIT) methods, the DPE method does not rely on precise adjustments of the hydrophilic–lipophilic balance (HLB) or the use of carefully balanced surfactant mixtures. Furthermore, unlike spontaneous emulsification techniques, the DPE method eliminates the need for organic solvents, making it a more versatile approach.^10^

This method typically requires an alkyl polyol as a fourth component to produce oil-in-water (O/W) emulsions and form the so-called D-phase.^11^ The presence of an alkyl polyol has been considered essential for preventing the formation of liquid crystals and for modifying the surfactant’s cloud point in the D-phase method.^11^ However, Kunieda challenged the necessity of alkyl polyols in forming this structure, suggesting they are not indispensable for achieving a successful formulation.^6^ Despite this ongoing debate, the DPE method demonstrates significant potential for the preparation of nanoemulsions, particularly those based on vegetable oils, due to its capacity for high lipid loading.^8,12,13^

Borage oil, a source of essential fatty acids, is well-known for its skin-beneficial properties, including anti-inflammatory effects. It is particularly enriched with ω-6 essential fatty acids (EFAs), such as gamma-linolenic acid (GLA).^14–16^ Typically, GLA is metabolized into dihomo-gamma-linolenic acid (DGLA) and arachidonic acid, both playing significant roles in maintaining skin health.^17^ The initial conversion of linoleic acid to GLA, catalyzed by the enzyme delta-6-desaturase, is impaired in individuals with atopic dermatitis (AD).^18^ Oral supplementation with borage oil has been shown to elevate GLA levels, thereby improving skin health.^19^

This study aims to develop a borage oil nanoemulsion using the D-phase emulsification method without the inclusion of an alkyl polyol component. The research encompasses an evaluation of emulsification conditions, statistical analyses, formulation optimization, morphological and toxicity assessments, as well as stability analyses of the resulting nanoemulsion.

## 2 Material and Method

### 2.1 Material

The materials used comprised polysorbate 80 (J.T. Baker, São Paulo, Brazil), ultrapure water (Milli-Q Plus—Gradient 10 system, Millipore), and borage oil (Sabará Químicos e Ingredientes S.A., Santa Bárbara d’Oeste, São Paulo, Brazil).

### 2.2 DPE Nanoemulsion Development without the Alkyl Polyol Component

The nanoemulsion preparation was conducted following the methodology described by Masiero et al.^8^ The formulation utilized borage oil and polysorbate 80 as its main components. The quality target product profile (QTPP) was established with the following specifications: an average hydrodynamic diameter (AHD) ranging from 100 to 500 nm, a polydispersity index (PdI) of less than 0.300, a zeta potential (ZP) below −20 mV, and physical stability under conditions of 30 °C ± 2 °C and 75% ± 5% relative humidity for a duration exceeding six months.

#### 2.2.1 Nanoemulsion Preparation

The nanoemulsion was prepared utilizing the surfactant and an initial water phase to form the D-phase, with borage oil as the lipid phase, at 25.0 ± 0.5 °C. The required final water volume to achieve the nanoemulsion was calculated based on the oil concentration, which ranged from 25.0% to 50.0% w/w. The formulations were prepared within a total of 50 g.

Initially the D-phase was homogenized under agitation at 300 rpm (Figure 1). Borage oil was then gradually incorporated, forming a gel-like structure (O/D gel). After the complete addition of the oil, the O/D gel was stirred for 20 min. The remaining water was then gradually added to the system, completing the emulsification process. Finally, the resulting oil-in-water (O/W) nanoemulsion was maintained under constant agitation at 300 rpm for an additional 30 min to ensure stability and homogeneity.

**Figure 1:**
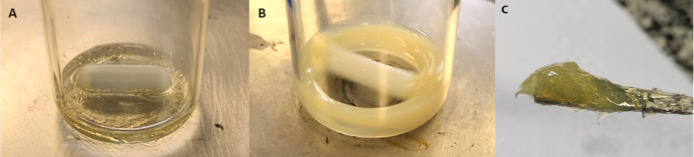
Preparation of borage oil nanoemulsion through the low-energy D-phase emulsification method. (A) D-phase composed of polysorbate 80 and initial water, showing a clear and homogeneous aspect. (B) Appearance of the D-phase after homogenization, exhibiting a turbid and opaque aspect, indicating the progression of the initial mixing process. (C) The O/D gel, characteristic of the process, formed after the addition of borage oil.

#### 2.2.2 Box–Behnken Design of Experiment

The influence of the nanoemulsion components on the average hydrodynamic diameter (AHD) was examined utilizing response surface methodology, specifically through the application of the Box–Behnken design with three factors at three levels (+1, 0, −1).^20^ A total of 15 formulations were prepared, including 3 central points (formulations 4, 11, and 15) as presented in Table S1. The independent variables in this study were the concentrations of borage oil (25.0%, 37.5%, 50.0% w/w), polysorbate 80 (3.5%, 4.0%, 4.5% w/w), and initial water (1.00%, 1.25%, 1.50% w/w). The dependent variable, or response, was the AHD. The experimental matrix for this study was generated using Minitab 17 statistical software (State College, PA, USA).

### 2.3 Optimization and Model Validation

The statistical software Minitab was utilized to conduct response optimization through the desirability function approach.^21^ The aim was also to achieve an average hydrodynamic diameter (AHD) of less than 500 nm.

After the optimization procedure, a new formulation was developed. To assess the adequacy of the mathematical model, both the observed and predicted AHD values for the nanoemulsions were determined.

### 2.4 Nanoemulsion Preparation on an Upscaled Batch

A larger batch of 1 kg was prepared, using a mechanical stirrer (IKA, RW20 digital), comprising the same components and parameters described in Section 3.1.1, with adjustments. Borage oil was added uniformly and in a controlled flow rate of 10 mL per minute, using a peristaltic pump (Milleto Biotecnologia), to ensure consistent incorporation of the lipid phase into the aqueous phase. Once the addition of borage oil was complete, the O/D phase was maintained under constant agitation for 20 min. The remaining water was added to complete the final nanoemulsion. This method was performed 3 times (*n* = 3).

### 2.5 Average Hydrodynamic Diameter and Polydispersity Index

The AHD and PdI of the droplets were assessed using dynamic light scattering. The samples were diluted at a 1:100 ratio with Milli-Q water, and the scattered light, emitted from a solid-state laser at 670 nm, was measured at a 90° angle.^22^ These measurements were performed using a Malvern Zetasizer Nano ZS90 instrument (Malvern Instruments, UK). During the testing period, the nanoemulsions were maintained at a temperature of 25 °C. The AHD was calculated using the Stokes–Einstein eq (eq 1), where *D* refers to translational diffusion coefficient (m^2^/s), *k*_B_ is the Boltzmann constant (1.38 × 10^–23^ J/K), *T* is the absolute temperature (K), η is the dynamic viscosity of the solvent (Pa s), and *r*_h_ is the hydrodynamic radius of the particle (m). While the PdI was determined as the standard deviation of the particle size distribution (σ) divided by the average particle diameter (2*a*), as specified in eq 2. Both AHD and PdI values represent the averages of three individual measurements.

1 $$D=\frac{k_{B}T}{6\pi \eta r_{h}}$$

2 $$PdI={\left(\frac{\sigma }{2a}\right)}^{2}$$

### 2.6 Zeta Potential

The zeta potential (ZP) was measured using a Malvern Zetasizer Nano ZS90 instrument (Malvern Instruments, UK) through the electrophoretic mobility method. The electrophoretic mobility values were subsequently converted into zeta potential (ζ) in millivolts (mV) using the Henry equation (eq 3). Prior to measurement, the samples were diluted in ultrapure Milli-Q water.^23^

3 $$U_{E}=\frac{2_{E}\zeta }{3_{\eta }}f\left(ka\right)$$

η and *E* refer to the viscosity and dielectric constant, respectively, of the solvent at 25 °C. UE represents the electrophoretic mobility, while ζ signifies the zeta potential. The applied field strength was set at 20 V/cm. The conductivity range assessed was 50 μS/cm, using a 0.9% (w/v) NaCl solution. The pH value was adjusted to 5.5 ± 0.2.

### 2.7 Physical Stability

The physical stability assay for the optimized formulations was carried out by storing the nanoemulsions in a climatic chamber (Climacell, MMM Group) maintained at 30 °C ± 2 °C and 75% ± 5% relative humidity. Evaluations were conducted weekly for the first 4 weeks, followed by assessments every three months over a one-year period.^24^ The critical quality attributes assessed included the average hydrodynamic diameter (AHD), polydispersity index (PdI), and zeta potential (ZP).

### 2.8 Microscopy

#### 2.8.1 Dark-Field Microscopy

Dark-field microscopy was performed using a CytoViva system (CytoViva Inc., Auburn, AL, USA), integrated into an Olympus BX51 upright microscope (Olympus, Tokyo, Japan). This setup was equipped with a CytoViva enhanced dark-field condenser and a liquid-core optical fiber illumination system. Hyperspectral images were acquired using a 60× oil-immersion objective lens.^25,26^

#### 2.8.2 Transmission Electron Microscopy

Transmission electron microscopy (TEM) images were acquired using a JEOL JEM-2100 microscope (JEOL, Japan), operating at an acceleration voltage of 200 kV. For sample preparation, uranyl acetate was used to enhance contrast by negatively staining the sample. The preparation process involved depositing 4 μL of the sample onto a 400-mesh copper grid coated with an ultrathin carbon film. The sample was allowed to rest for 4 min, and the excess liquid was carefully removed using filter paper. Subsequently, 4 μL of a 2% (w/w) uranyl acetate solution was applied to the same grid area. The uranyl acetate solution was left in contact with the grid for 2 min, after which the excess was removed with clean filter paper.^27^

### 2.9 X-ray Diffraction

XRD measurements of borage oil gels with and without glycerin were performed on a Rigaku (Japan) Miniflex 300 X-ray diffraction meter equipped with a Cu source (λ = 1.5418 Å), operating at 30 kV and 15 mA in the 10 to 90° (2θ) range, with a 0.02° step and a counting time of 1 s per step. The equipment features a parallel beam geometry and a high-sensitivity scintillation detector, suitable for analyzing gel samples. The equipment was equipped with a standard sample holder for analyzing powders and flat solids, coupled with a rotating sample holder, which promotes better statistical diffractogram acquisition, reducing the effects of particle orientation and improving the representativeness of the data obtained.^28^

### 2.10 Toxicity Assay Using the *Galleria Mellonella* L. Model

The toxicity assay using *Galleria mellonella* L. larvae was conducted following the method described by Masiero et al.,^8^ with modifications. For the experiment, larvae measuring 2.0 to 2.5 cm in length and with an average weight of 160 mg were maintained at 28 °C. A 20 μL aliquot of the pure, undiluted sample (50% w/w borage oil nanoemulsion) was injected into the last left proleg. Larvae receiving the test solutions were compared to a control group that was injected with PBS (pH 7.4) only. Groups of 20 larvae were incubated at 35 °C and monitored daily for 5 days to evaluate toxicity, survival profile, and health index, according to the established criteria (Table 1). The larval survival curves were compared using the Log-rank (Mantel–Cox) test, while the health index was analyzed using a two-way ANOVA (*p*-value <0.05).

**Table 1:** Health Index Scoring for *G. mellonella* Larvae (Adapted from Loh et al.^29^)­^a^

category	description	score
activity	no movement	0
	minimal movement in response to stimulus	1
	movement when stimulated	2
	movement without stimulus	3
cocoon formation	no cocoon	0
	partial cocoon	0.5
	full cocoon	1
melanization	black larva	0
	black larva with brown spots	1
	≥3 spots on beige larva	2
	<3 spots on beige larva	3
	no melanization	4
survival	dead	0
	alive	2

a Interpretation: healthy larvae (uninfected) exhibit a total score of 9–10.

## 3 Result and Discussion

### 3.1 Nanoemulsion Preparation

Figure 2 shows the D-phase (Figure 2A), which exhibited rapid dispersion of the borage oil upon agitation, forming the O/D gel (Figure 2B). Initially, the O/D gel appeared transparent and fluid; however, with an increase in borage oil concentration, it became turbid and demonstrated higher viscosity. Contrary to Sagitani’s assertion regarding the challenges of incorporating high oil concentrations into such structures,^11,12^ the borage oil was seamlessly integrated into the system without any evidence of phase separation. Upon the gradual addition of the remaining water, the fine oil droplets from the O/D gel dispersed, forming oil-in-water (O/W) nanoemulsion (Figure 2C). The rapid dispersion of the oil phase forming the O/D gel, can be attributed to the low interfacial tension between the oil and the surrounding medium.^12^ This phenomenon confirms the presence of borage oil in the O/D gel, consistent with the methodology described by Endoo and Sagitani.^30^ In that study, the authors suggested that the resulting gel should be transparent and fluid, avoiding the formation of liquid crystals. However, Kunieda disputed this assertion, arguing that the transparent gel observed by Sagitani was, in fact, a liquid crystal.^6^ The gel-like structure forms through four distinct phases: micellar solution, micellar cubic, hexagonal liquid crystal, and lamellar liquid crystal, with the micellar cubic phase being critical for the O/D gel formation, which transitions into the final liquid O/W emulsion.^6^ Although the alkyl polyol is not essential for the formation of the gel, its inclusion is intended to modify the surfactant cloud point and reduce the gel viscosity, thereby facilitating emulsion preparation.^10^ The subsequent addition of the remaining water readily dispersed the O/D gel, resulting in the final borage oil nanoemulsion with a milky appearance. This was achieved with the content of 50% (w/w) borage oil and 4% (w/w) polysorbate 80, without hydrophilic–lipophilic balance (HLB) adjustment or the inclusion of an alkyl polyol component.

**Figure 2:**
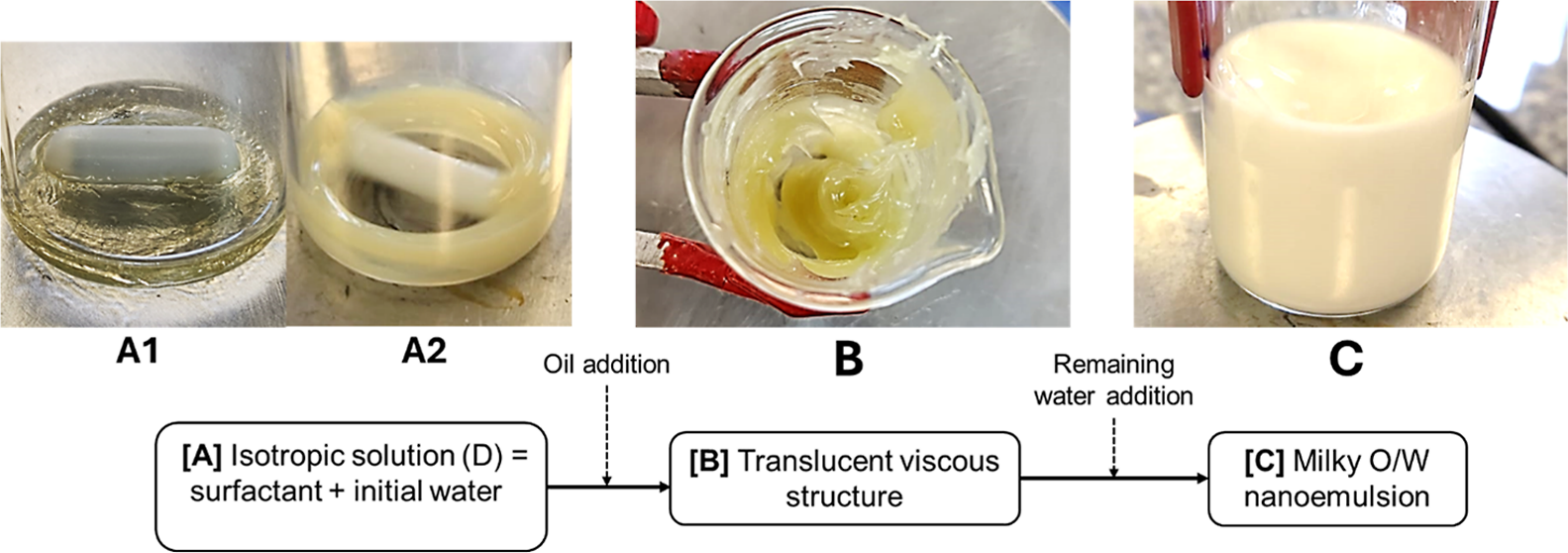
Sequential stages of the formulation process: (A1) shows the initial mixture of surfactant and water before agitation. (A2) Presents the D-phase obtained after agitation. (B) Shows the formtion of the O/D gel after the oil addition. (C) Illustrates the final nanoemulsion formed after the incorporation of the remaining water.

#### 3.1.1 Box–Behnken Design of Experiment

A total of 15 experiments were performed employing Box–Behnken to assess the influence and interactions of the independent variables—borage oil concentration, surfactant concentration, and initial water concentration—on the AHD, the dependent variable. The AHD ranged from 257.8 to 505.1 nm, with PdI values between 0.036 and 0.183, indicating a narrow particle size distribution (Table 2). ZP values were between −42.7 and −23.0 mV.

**Table 2:** AHD and PdI via DPE Process without Alkyl Polyol^a^

formulation	BO	T80	H_2_O_*i*_	AHD (nm)	PdI
1	25.0	4.0	1.00	264.3 ± 0.2	0.070 ± 0.025
2	37.5	4.5	1.50	279.7 ± 3.7	0.065 ± 0.034
3	37.5	4.5	1.00	-	-
4	37.5	4.0	1.25	312.6 ± 4.4	0.036 ± 0.025
5	37.5	3.5	1.50	403.8 ± 3.3	0.174 ± 0.036
6	50.0	4.0	1.00	-	-
7	50.0	4.5	1.25	505.1 ± 23.3	0.122 ± 0.104
8	25.0	4.0	1.50	257.8 ± 3.9	0.141 ± 0.059
9	37.5	3.5	1.00	453.8 ± 2.6	0.146 ± 0.035
10	25.0	3.5	1.25	391.7 ± 4.6	0.163 ± 0.017
11	37.5	4.0	1.25	339.9 ± 8.6	0.140 ± 0.021
12	25.0	4.5	1.25	280.7 ± 4.3	0.050 ± 0.061
13	50.0	4.0	1.50	447.1 ± 6.2	0.141 ± 0.066
14	50.0	3.5	1.25	313.3 ± 0.8	0.183 ± 0.088
15	37.5	4.0	1.25	355.8 ± 2.8	0.135 ± 0.043

a BO: Borage oil; T80: polysorbate 80; H_2_O_*i*_: initial water; AHD: average hydrodynamic diameter; PdI: polydispersity index; ZP: zeta potential.

The analysis of variance (ANOVA) presented in Table 3 demonstrates the statistical significance and robustness of the quadratic regression model used to evaluate the effects of independent variables and their interactions on the AHD of the nanoemulsion. The model achieved a highly significant *p*-value (<0.001; α = 0.05), indicating that the independent variables and their interactions significantly influence the response variable. This finding is further supported by the high coefficient of determination (*R* ^2^ = 97.48%), which shows that the model accounts for most of the variability in the AHD. Additionally, the adjusted *R* ^2^ (94.95%) confirms the model’s robustness by accounting for the number of predictors, while the predicted *R* ^2^ (84.17%) reflects its ability to predict new responses with reasonable accuracy.

**Table 3:** ANOVA and Quadratic Regression of Different Adjusted Response Models, for the AHD of the Nanoemulsion

source	DF	SQ (Aj.)	QM (Aj.)	*F* value	*P*-value
model	7	459,017	65,574	38.64	0.001
linear	3	312,891	104,297	61.46	0.001
BO	1	135,408	135,408	79.79	0.001
T80	1	42,413	42,413	24.99	0.002
H_2_O_*i*_	1	135,070	135,070	79.59	0.001
square	1	53,883	53,883	31.75	0.001
H_2_O_i_*H_2_O_*i*_	1	53,883	53,883	31.75	0.001
interaction	3	92,243	30,748	18.12	0.001
BO*T80	1	22,952	22,952	13.52	0.008
BO*H_2_O_*i*_	1	13,995	13,995	8.25	0.024
T80*H_2_O_*i*_	1	55,296	55,296	32.58	0.001
error	7	11,879	1697		
lack-of-fit	5	10,932	2186	4.61	0.188
pure error	2	948	474	*	*
total	14	470,897			
summary of the model	*R* ^2^		*R* ^2^ (adj)		*R* ^2^ (pred)
	97.48%		94.95%		84.17%

The linear terms for borage oil concentration (BO), surfactant concentration (T80), and initial water concentration (H_2_O_*i*_) exhibit statistical significance (*p*-value <0.01; α = 0.05), with initial water concentration (H_2_O_*i*_) showing the most substantial impact. This indicates that initial water concentration is a key factor in controlling the particle size of the nanoemulsion. Moreover, the quadratic term for initial water concentration (H_2_O_i_*H_2_O_*i*_) is also significant (*p*-value <0.001; α = 0.05), suggesting a nonlinear relationship between this variable and the AHD. This nonlinear effect underscores the importance of optimizing the initial water concentration to achieve the desired nanoemulsion properties. The interaction terms BO*T80, BO*H_2_O_*i*_, and T80*H_2_O_*i*_ are statistically significant. This result highlights the critical role of variable interactions in determining the AHD, emphasizing that the combined effects of these parameters are as important as their individual contributions. The lack-of-fit was nonsignificant (*p*-value = 0.188; α = 0.05), indicating that the model adequately fits the experimental data and that no substantial variability remains unexplained.

#### 3.1.2 Regression Equation for the Variables

The eq 4 describes the AHD, considering the linear, quadratic, and interaction terms. The linear coefficients indicate the direct influence of each component on the particle size. For instance, the negative coefficient of −14.4 for borage oil (BO) suggests that an increase in BO concentration is associated with a decrease in particle size, whereas the positive coefficient of +867 for polysorbate 80 (T80) indicates that an increase in T80 concentration results in an increase in particle size. Furthermore, the presence of the quadratic term +1922 H_2_O_i_*H_2_O_*i*_ reveals a nonlinear relationship between the initial water concentration (H_2_O_*i*_) and particle size, indicating that increasing the H_2_O_*i*_ concentration leads to an increase in particle size. The coefficient value (1922) has a significant impact on the particle size. Interaction terms, such as +12.12 BO*T80 and −18.93 BO*H_2_O_*i*_, illustrate how combinations of different components influence particle size, either increasing or decreasing it. For example, a positive coefficient of +12.12 for BO*T80 indicates that simultaneous increases in BO and T80 concentrations lead to an increase in particle size, while a negative coefficient of −18.93 for BO*H_2_O_*i*_ indicates that increasing both BO and H_2_O_*i*_ concentrations results in a reduction in particle size. These effects are illustrated in the response surface and contour plots (Figure 3).

4 $$AHD\;\left(nm\right)=-735-14.4\;BO+867\;T80-853\;H_{2}O_{i}+1922\;H_{2}O_{i}*H_{2}O_{i}+12.12\;BO*T80-18.93\;BO*H_{2}O_{i}-941\;T80*H_{2}O_{i}$$

**Figure 3:**
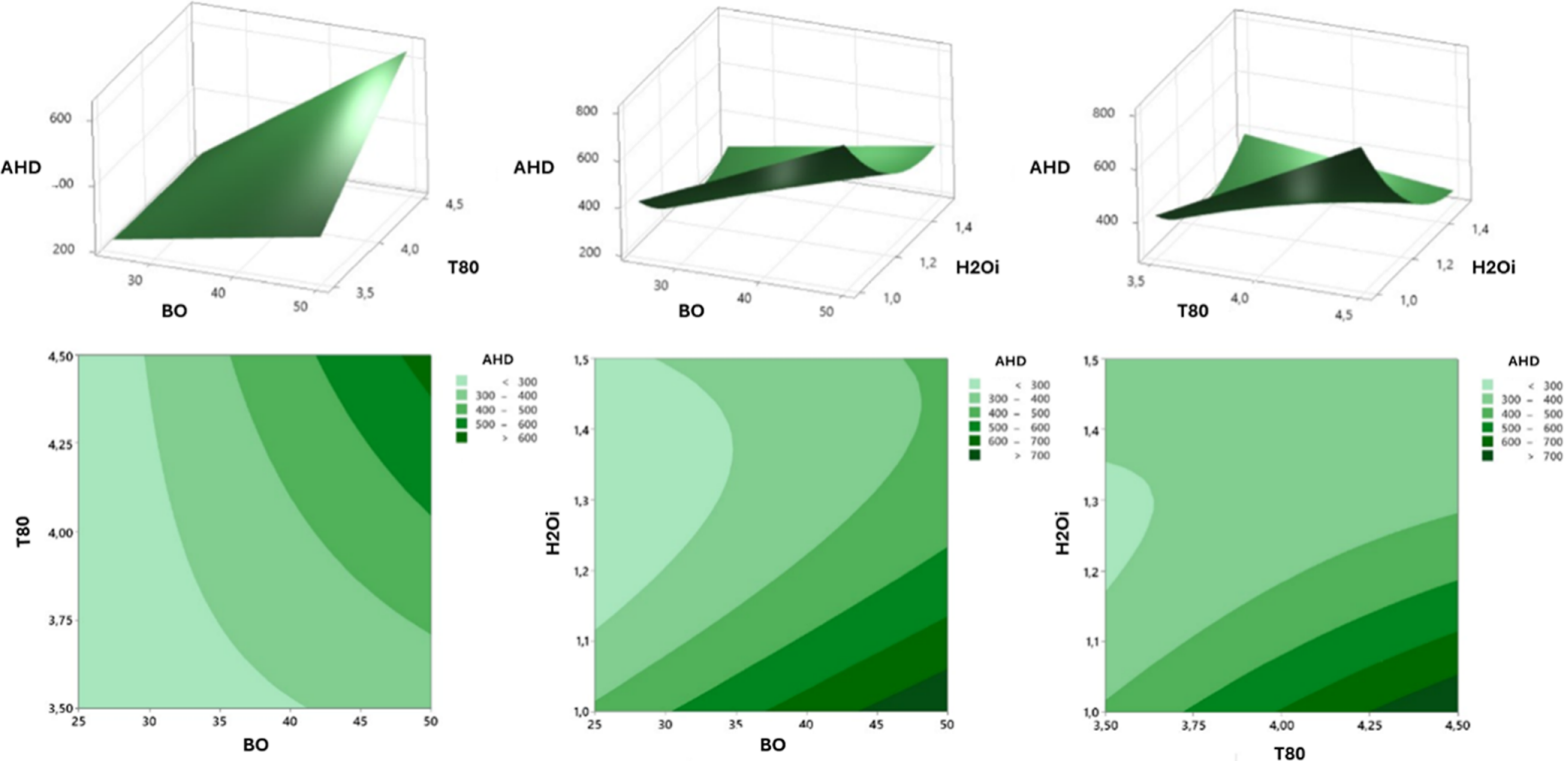
Response surface and contour plot for the evaluation of AHD of borage oil nanoemulsion, considering the variables borage oil concentration, polysorbate 80, and initial water.

#### 3.1.3 Preparation of the Nanoemulsion on an Upscaled Batch

Following the method outlined in Section 3.1, the nanoemulsion was prepared on a larger scale to evaluate the feasibility of scaling up the process for potential commercial application. During the addition of borage oil, the initially transparent gel structure gradually turned turbid, similarly as observed in the laboratory scale (Figure 4A). Subsequently, the remaining water was added. This step facilitated the dispersion of fine oil particles from the internal phase into the aqueous continuous phase, ultimately resulting in the formation of the final oil-in-water (O/W) nanoemulsion (Figure 4B). The AHD for the 3 batches were: 339.2 ± 3.8, 380.8 ± 4.7, and 350.5 ± 2.4; PdI of 0.118 ± 0.090, 0.200 ± 0.081, and 0.129 ± 0.068; and ZP of −35.7 ± 0.6, −41.0 ± 1.9, and −38.4 ± 4.8.

**Figure 4:**
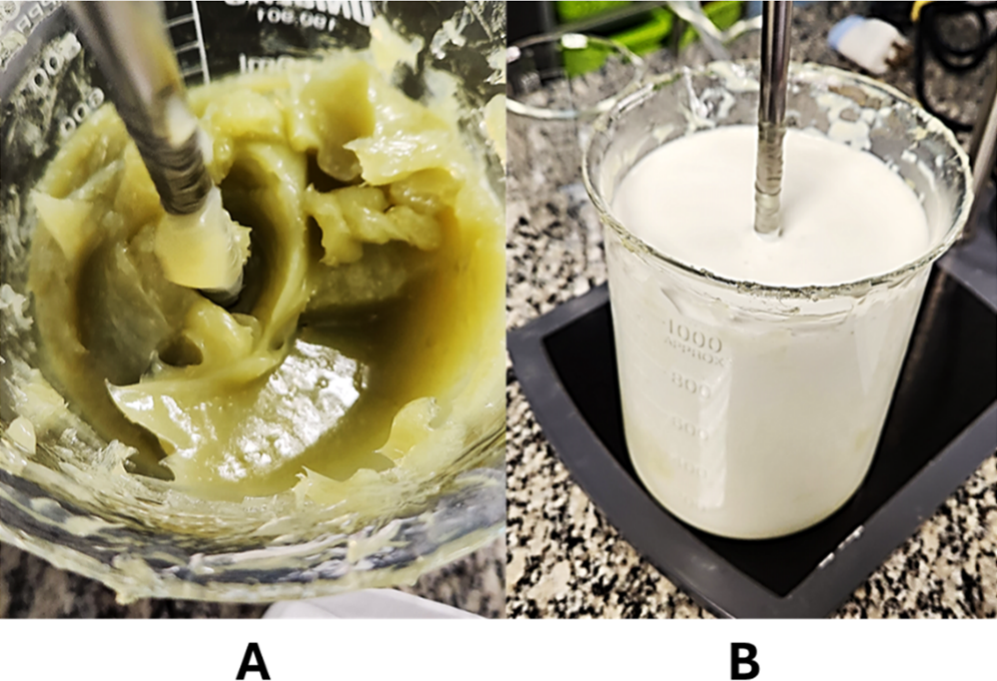
Visual aspects of the DPE process at a temperature of 25 °C prepared on a large scale. (A) Represents the gel-like structure, after the oil addition. (B) Shows the final white opaque nanoemulsion.

### 3.2 Optimization and Model Verification

The optimal conditions, as determined by the desirability function (1.0), were achieved by combining a borage oil concentration of 31.0% (w/w), a surfactant concentration of 3.8% (w/w), and an initial water concentration of 1.5% (w/w) (Figure 5). This combination yielded a theoretical AHD of 350.0 nm. The experimental and theoretical AHD are shown in Table 4, verifying the proposed model.

**Figure 5:**
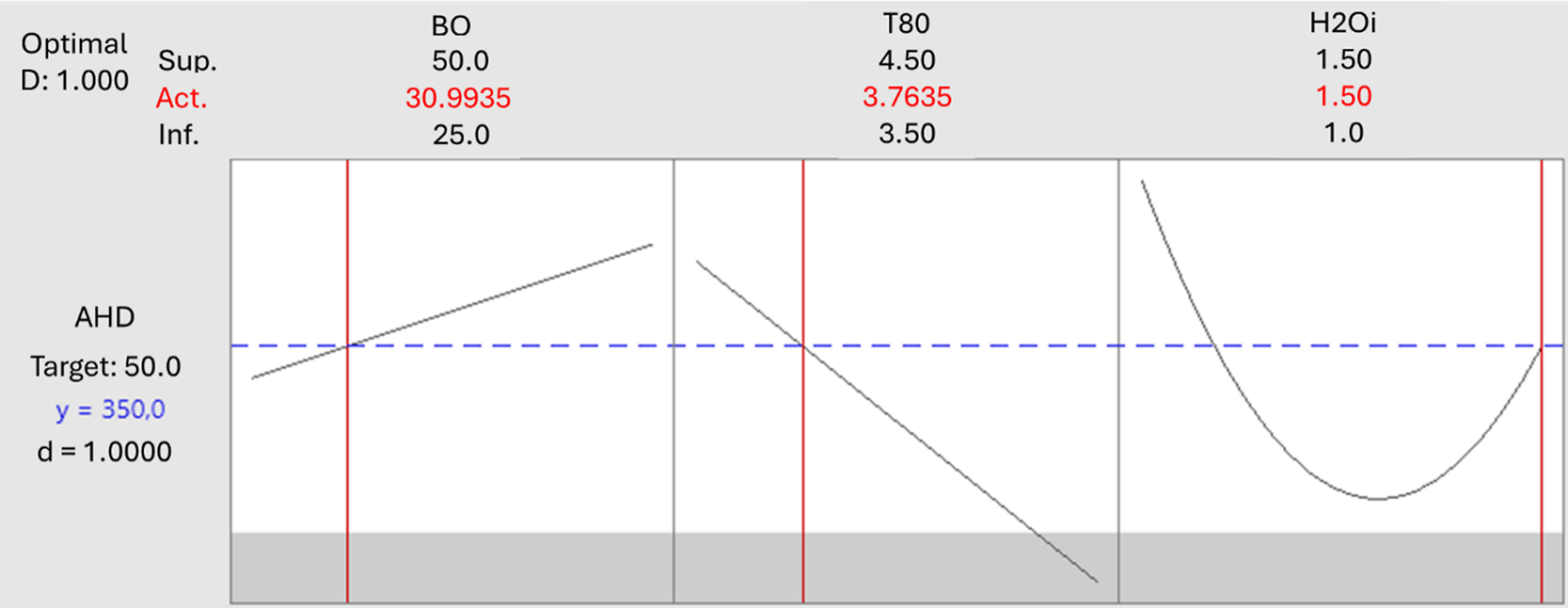
Main effects plots for AHD as a function of components and preparation variables by the DPE process.

**Table 4:** Theoretical and Experimental Values for the AHD of Particles in the Optimized Formula

AHD experimental (nm)	AHD theoretical (nm)	95% CI	PdI
300.5 ± 2.2	350.0	284.6–415.4	0.25 ± 0.004
232.4 ± 4.5	250.0	171.2–328.8	0.13 ± 0.024
422.4 ± 8.1	450.0	399.1–500.9	1.19 ± 0.076

### 3.3 Physical Stability

The physical stability of nanoemulsions is a critical aspect in pharmaceutical development, particularly due to the challenge posed by Ostwald ripening, which can occur over prolonged periods.^31^ Ostwald ripening refers to the phenomenon where small droplets progressively decrease in size while larger droplets increase, eventually leading to the complete dissolution of small droplets into the continuous phase during storage.^32^ In our study, polysorbate 80 was selected as the surfactant to promote stabilization. For the DPE method, the AHD immediately after preparation was 300.5 ± 2.2 nm, and the final AHD after 12 months was 339.9 ± 5.2 nm. These results indicated that the preparation remained stable over the 12-month evaluation period, with no signs of phase separation. The initial zeta potential value was −59.4 ± 3.8 mV immediately after preparation and −29.1 ± 3.1 mV after 12 months.

### 3.4 Microscopy

#### 3.4.1 Dark Field Microscopy

The morphological analysis of the nanoemulsion revealed the presence of particles dispersed with a uniform distribution across the field of view (Figure 6). The observed particles exhibit a spherical shape and are visibly reflective, which is characteristic of the dark field technique. The contrast generated between the particles and the dark background highlights the presence of bright spots, suggesting that the particles are well-dispersed in the medium, with no signs of aggregation or coalescence. The presence of spherical particles and their uniform distribution indicate a well-structured and stable system. The analysis suggests that the nanoemulsion is well-suited for these applications, offering effective control over particle size and system stability.

**Figure 6:**
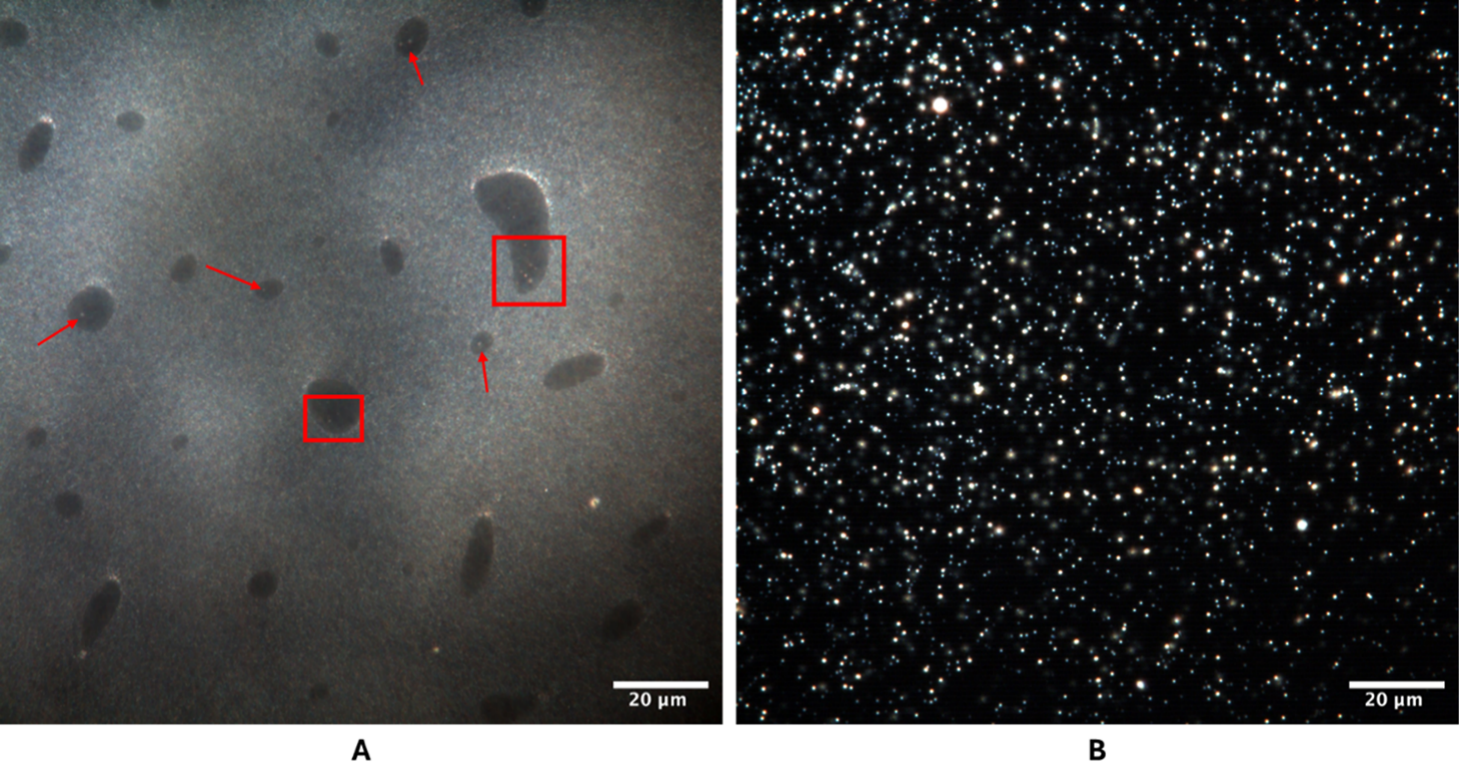
Dark field microscopy images captured by the CytoViva System. (A) Shows the O/D gel, where water pockets were observed, containing nanometric droplets (indicated in red). (B) Illustrates the O/W nanoemulsion, the different sizes of the particles correspond to the different layers in the image.

#### 3.4.2 Transmission Electron Microscopy

The morphological analysis was conducted using transmission electron microscopy (TEM), revealing the presence of spherical nanometric droplets with a uniform distribution across the field (Figure 7). The droplets exhibit sharp edges and well-stabilized interfaces, suggesting the effective action of surfactants in the formulation. According to the guidelines established by the Food and Drug Administration (FDA), particle size analysis in nanoemulsions is recommended to be conducted using at least two distinct methods to ensure the accuracy and reproducibility of results.^33^ In addition to TEM, the DLS analysis previously performed showed significant agreement between the methods, with both techniques indicating an AHD of approximately 300 nm. This consistency reinforces the reliability of the measurements and highlights the robustness of the system in terms of particle size control.

**Figure 7:**
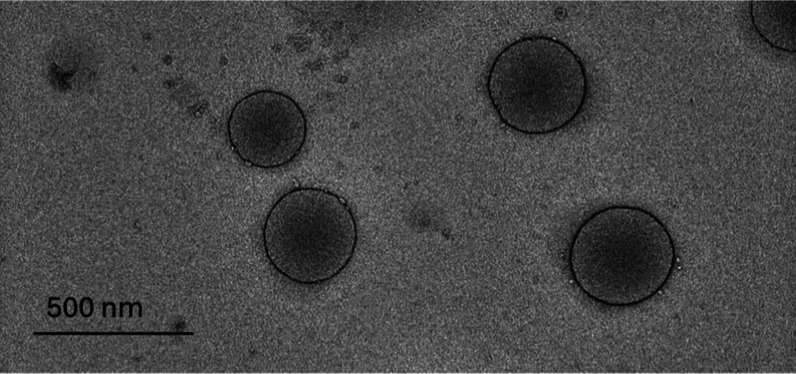
TEM image of the borage oil nanoemulsion on copper grid coated with an ultrathin carbon film.

### 3.5 X-ray Diffraction

Analysis of the X-ray diffraction (XRD) patterns of the O/D gel formulations (Figure 8) with (C) and without polyol (S), reveal characteristics of materials with low ordering over long distances (crystallinity). All compositions exhibited a broad and intense peak near 2θ ∼ 20°, corroborating the formation of poorly organized domains (essentially amorphous), and without the multiple sharp peaks typical of conventional crystalline materials.

**Figure 8:**
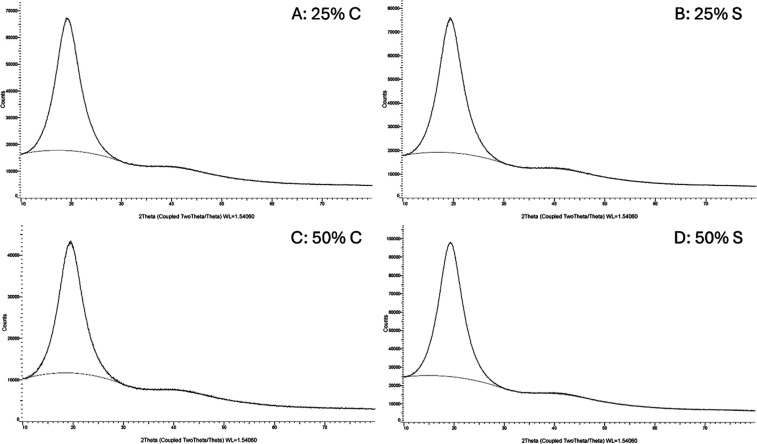
X-ray diffraction patterns of borage oil gels with and without polyol.

Glycerin, recognized for its wetting properties, can weaken liquid crystalline microstructures by acting as a competitive solvent for residual water, thus promoting greater disorganization between the polar chains. However, this is not apparently observed in the samples, since the breakdown of organization is associated with short distances, thus demonstrating that the general crystallinity characteristics of the materials are not significantly affected by the presence of glycerin. The absence of glycerin in these formulations may favor a greater degree of order within the liquid crystalline domains, consistent with theoretical expectations for oil-rich systems. These findings clarify the disruptive effect that glycerin can exert, even under conditions favorable to supramolecular organization, and contribute to the rational design of functional gels for pharmaceutical applications.

### 3.6 Toxicity Assay Using the *G. Mellonella* L. Model

The assay for borage oil nanoemulsion was conducted and did not show significant toxicity among the groups tested (Figure 9).

**Figure 9:**
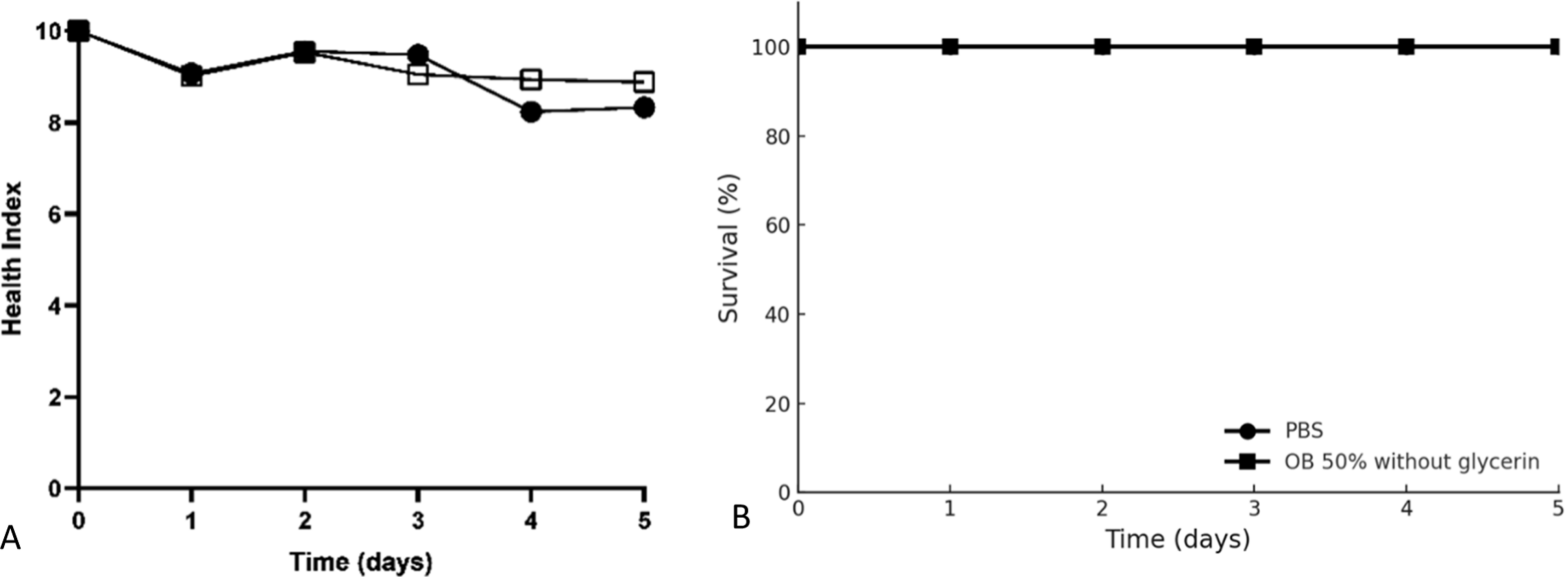
Toxicity assay of borage oil nanoemulsion prepared by the DPE method in the invertebrate model *G. mellonella* L. Health index (A) and survival rate (B) of *G. mellonella* L. larvae injected with PBS: phosphate-buffered saline, pH 7.4; 50% Borage oil nanoemulsion.

## 4 Conclusion

A stable borage oil nanoemulsion with a high oil concentration and an average hydrodynamic diameter (AHD) of 300–400 nm was obtained using low surfactant content through the DPE process. The use of statistical tools revealed surprising phenomena, not predictable and of fundamental importance for the proper obtention of the nanoemulsion. The removal of polyol for the successful development of a new formulation was found to be a unique, surprising condition, not reported in the literature to date. In this formulation, it was surprising to observe that the initial water was the statistically most significant variable for AHD reduction. Additionally, this low-energy method allowed the scale-up without specific mechanical devices.

## Supplementary Material



## Notes

Box–Behnken design of experiments for evaluation of average hydrodynamic diameter and polydispersity index (Table S1); thermogravimetric analysis (TGA/DTA) and differential scanning calorimetry (DSC) methods and results for borage oil gel formulations with and without glycerin (Figures S1 and S2); stability evaluation of optimized and maximum-concentration formulations at laboratory and scale-up levels, including composition (Table S2) and long-term AHD, PdI, and zeta potential data after six and 12 months (Table S3) (PDF)
