# Supplementary material for: High-Load Borage Oil Nanoemulsion Development via Polyol-Free D‑Phase Emulsification
Source: ACS Omega. 2026 Jan 30;11(6):10451–60. doi: 10.1021/acsomega.5c11660 (PMC12917694; doi:10.1021/acsomega.5c11660)
Supplement: Supplementary file 1 [file ao5c11660_si_001.pdf]

Supporting Information for

High-Load Borage Oil Nanoemulsion Development via Polyol-Free D-Phase Emulsification

*Jéssica Fagionato Masiero, Jonnatan Julival Santos, Andriéli Bacega, Enzo Boniconte Santomartino, Luiza de Oliveira Macedo, Geraldo José Arantes, Gabriel Lima Barros de Araújo, Kelly Ishida, Nádia Araci Bou-Chacra**

1 METHOD

1.1. BOX-BEHNKEN DESIGN OF EXPERIMENT

Table S1 – Box-Behnken experiment for evaluation of average hydrodynamic diameter and polydispersity index.

**Formulation**	**BO**	**T80**	**H_2O_i**
1	25.0	4.0	1.00
2	37.5	4.5	1.50
3	37.5	4.5	1.00
4	37.5	4.0	1.25
5	37.5	3.5	1.50
6	50.0	4.0	1.00
7	50.0	4.5	1.25
8	25.0	4.0	1.50
9	37.5	3.5	1.00
10	25.0	3.5	1.25
11	37.5	4.0	1.25
12	25.0	4.5	1.25
13	50.0	4.0	1.50
14	50.0	3.5	1.25
15	37.5	4.0	1.25

BO: Borage oil; T80: Polysorbate 80; H_2O_i: Initial water.

1.2. THERMAL ANALYSIS

1.2.1. THERMOGRAVIMETRY

Thermogravimetry (TG) measurements were performed on a DTG-60 analyzer (Shimadzu, Japan). Samples of borage oil gels with or without glycerin (~10 mg) were added to platinum crucibles and heated in a controlled atmosphere of synthetic air (flow rate of 50 mL min^{-1}), in the temperature range of 25 to 900 °C, with a heating rate of 10 °C min^{-1}.

1.2.2. DIFFERENTIAL SCANNING CALORIMETRY

Thermal analyses were conducted using a DSC Q10 differential scanning calorimeter (TA Instruments, USA). Approximately 10 mg of sample were placed in hermetically sealed aluminum crucibles. The tests were performed in the temperature range from –80 °C to 170 °C, using a closed

alumina crucible, with gas atmosphere. The equipment's measurements allowed the determination of thermal transitions, such as melting, crystallization, and glass transition temperatures, providing information on the stability and thermal behavior of the materials.

2. RESULTS

2.1. THERMAL ANALYSIS

2.1.1. THERMOGRAVIMETRY

The thermogravimetric (TGA) and differential thermal analysis (DTA) (Figure S1) results for the 50C (with glycerin) and 50S (without glycerin) gel formulations elucidate the thermal stability profile and compositional differences between these oil-rich systems. Both samples exhibit an initial mass loss event up to approximately 170°C, which can be attributed to the evaporation of water, glycerin (when present), and other volatile constituents. The 50C sample presents a higher mass loss in this region (around 3.5%) compared to 50S (approximately 2%), reflecting the higher volatility and hygroscopicity of glycerin. The temperatures of the initial endothermic DTA events are close for both samples (around 169–172°C), suggesting a similar onset for the removal of these volatiles. At higher temperatures, both formulations display a major decomposition event commencing near 290°C, which corresponds to the thermal degradation of the organic matrix, predominantly borage oil and surfactant. The overall extent of mass loss is high for both systems (above 95%), indicating the primarily organic nature of these gels. The near-identical temperature for the main decomposition event and the similar residual masses emphasize that the inclusion of glycerin has minimal impact on the thermal stability of the overall system under high-temperature conditions.

Figure S1. Thermogravimetric and Differential Thermal Analysis of Borage Oil Gel Formulations

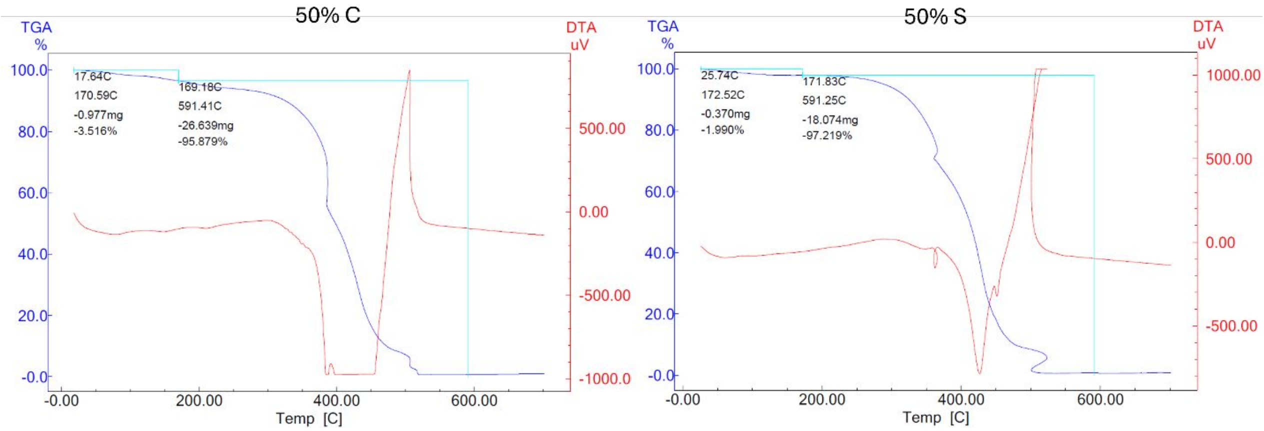

2.1.2. DIFFERENTIAL SCANNING CALORIMETRY

The differential scanning calorimetry (DSC) analyses for the gel formulations containing either (C) or not (S) glycerin (Figure S2) provide additional insights into the thermal transitions and microstructural organization of these systems. Both samples underwent heating, cooling, and a second heating cycle, allowing for the evaluation of thermal events such as melting, crystallization, and potential glass transitions.

Figure S2. Differential Scanning Calorimetry Thermograms of Borage Oil Gels With and Without Glycerin

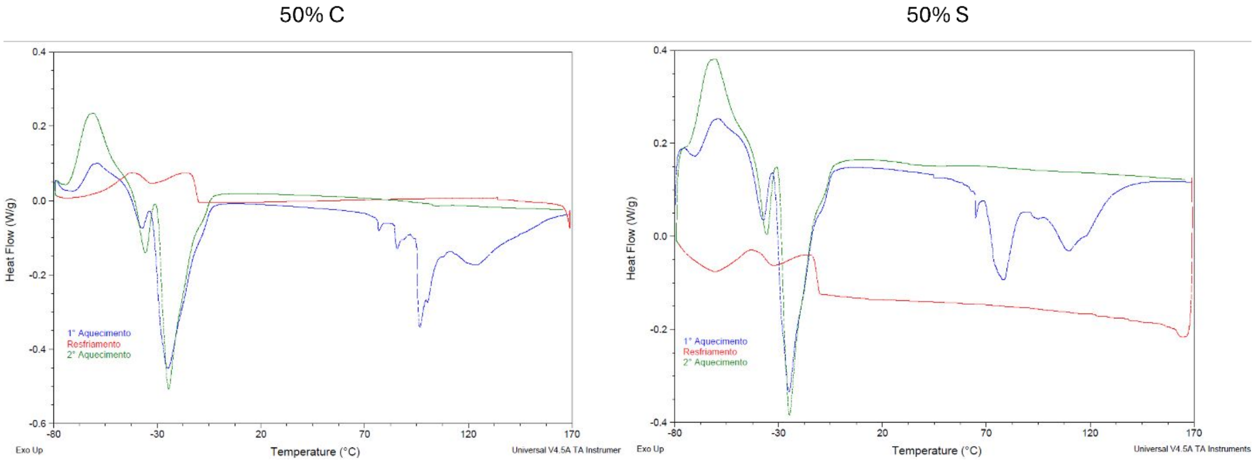

The thermograms reveal broad thermal events without sharp, well-defined melting or crystallization peaks within the analyzed temperature range (approximately -80°C to 170°C). This profile suggests a predominance of amorphous or liquid crystalline domains, corroborating the XRD results that indicated short-range order instead of long-range crystalline structures. The absence of prominent transitions in the cooling and second heating cycles also suggests that even

after thermal cycling, the systems do not reorganize to form highly crystalline or well-defined mesophases, highlighting the stability or persistence of their original supramolecular arrangement. A comparison between the two samples shows minor differences in baseline heat flow and event breadths, but fundamentally similar thermal behaviors. The incorporation of glycerin (sample C) does not introduce sharp new transitions but might contribute to subtle shifts in the baseline or broadened thermal events due to its plasticizing effect and its interaction with the polar domains of the system. This again is consistent with the TGA and XRD data, where the presence of glycerin affected the microstructure and volatility but did not significantly alter the main thermal stability framework.

3. OPTIMIZED FORMULATIONS IN STABILITY

With the aim of evaluating stability to achieve the longest possible shelf life and to facilitate scale-up transposition, one formulation was prepared in addition to the optimized ones, with higher oil and initial water concentration (Table S2). These formulations have been kept in a climatic chamber until the present moment and are being periodically evaluated. So far (twelve months – laboratory scale, six months – scale-up), they present AHD, PdI, and ZP according to Table S3.

Table S2 – Formulation developed at laboratory and scale-up levels, under stability evaluation.

**Components (g)**	**Laboratory (50 g)**		**Scale-up (1000 g)**
	**F.Opt.**	**F.Max.**	**F.Max.**
**BO**	15.50	25.0	500.0
**T80**	1.88	2.0	40.0
**H_2O_i**	0.75	1.0	20.0
**H_2O_f**	31.87	22.0	440.0

BO: Borage oil; T80: Polysorbate 80; H_2O_i: Initial water; H_2O_f: Final water; F.Opt.: Optimized formulation; F.Max.: Maximum concentration formulation.

Table S3 – AHD, PdI, and ZP of the formulation under stability evaluation after a period of six and twelve months.

	**Laboratory (12 months)**		**Scale-up (six months)**
	**F.Opt.**	**F.Max.**	**F.Max.**
**AHD (nm)**	339.9 ± 5.2	345.9 ± 8.3	345.4 ± 2.1
**PdI**	0.20 ± 0.06	0.21 ± 0.01	0.15 ± 0.02

**ZP (mV)**	-29.1 ± 3.1	-42.7 ± 6.2	-39.9 ± 0.2

F.Op.: Optimized formulation; F.Max.: Maximum concentration formulation; AHD: Average hydrodynamic diameter; PdI: Polydispersity index; ZP: Zeta potential.
